# Recurrent Embolic Stroke Caused by a Thrombosed Common Carotid Artery Aneurysm: An Uncommon and Initially Indeterminate Source of Embolism

**DOI:** 10.7759/cureus.90525

**Published:** 2025-08-19

**Authors:** Takahiro Kumagawa, Hiroshi Negishi, Shun Yamamuro, Katsunori Shijo, Atsuo Yoshino

**Affiliations:** 1 Neurological Surgery, Nihon University School of Medicine, Tokyo, JPN

**Keywords:** carotid artery stenting, embolic stroke of undetermined source, embolization, extracranial carotid artery aneurysm, repeated large vessel occlusion

## Abstract

Recurrent ischemic stroke with no clearly identified embolic source poses a significant diagnostic challenge. We report the case of a 73-year-old woman who experienced four episodes of cerebral infarction, all localized to the right middle cerebral artery territory. According to medical records from the referring hospital, the first three strokes occurred over three years. They were diagnosed as having an embolic stroke of undetermined source after extensive evaluation failed to identify a definitive cause. Although carotid ultrasonography and magnetic resonance angiography had revealed a right common carotid artery aneurysm during earlier evaluations, the absence of thrombus led clinicians to exclude it as an embolic source. At the time of the fourth stroke, follow-up carotid ultrasound demonstrated turbulent flow and bright, mobile intraluminal structures within the aneurysm, strongly suggesting thrombus formation. The patient subsequently underwent successful endovascular treatment with stent placement and coil embolization. No recurrence of stroke has been observed during the five-year follow-up. This case highlights that extracranial carotid artery aneurysms, although rare, should be considered as potential embolic sources, particularly when intra-aneurysmal thrombus is suspected. Endovascular intervention may be an effective strategy to prevent recurrent embolic stroke in such patients.

## Introduction

Extracranial carotid artery aneurysms (ECAAs) are exceedingly rare, accounting for less than 4% of all extracranial arterial aneurysms, and can result from atherosclerosis, trauma, infection, or congenital defects. While they may remain asymptomatic for years, thrombosed ECAAs pose a significant risk of cerebral embolism due to distal thrombus migration [1].

Embolic stroke of undetermined source (ESUS) is a subtype of cryptogenic stroke, and its primary causes include paroxysmal atrial fibrillation, cancer-associated embolism, arterogenic embolism, and paradoxical embolism [2,3]. In clinical practice, ESUS workups often emphasize prolonged cardiac monitoring and screening for paradoxical embolism. At the same time, structural vascular anomalies such as ECAAs may be overlooked, especially in the absence of thrombus or overt clinical signs like cervical mass or cranial nerve palsy.

Importantly, standard cardiac and intracranial imaging frequently fails to visualize cervical vascular lesions, contributing to the underdiagnosis of ECAAs in up to one-third of cryptogenic or ESUS-classified strokes [4]. Several case reports have indicated that such aneurysms are sometimes only recognized after recurrent embolic episodes or follow-up vascular imaging [5,6]. This highlights the importance of repeated and targeted vascular evaluations, such as carotid ultrasonography and magnetic resonance angiography (MRA), in patients with recurrent stroke of initially undetermined origin.

We present a case of recurrent right middle cerebral artery (MCA) territory infarctions, comprising two large vessel occlusions (LVOs) and two non-LVO events, initially classified as ESUS. A thrombosed common carotid artery (CCA) aneurysm was later identified as the likely embolic source. This case emphasizes the need to consider extracranial carotid aneurysms as a potentially underrecognized etiology in recurrent embolic stroke.

## Case presentation

**Figure 1:**
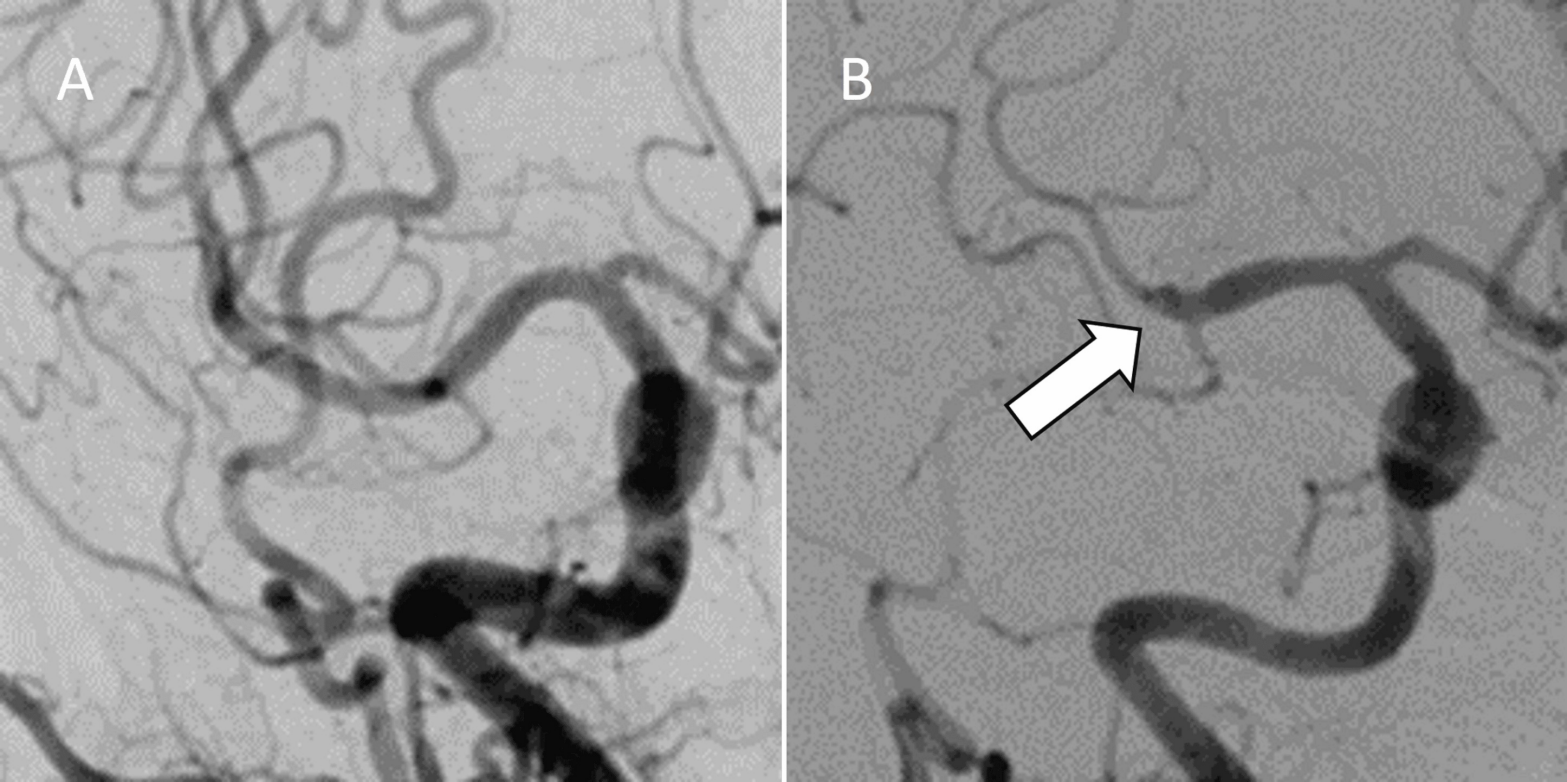
Cerebral angiography performed at the referring hospital (A) Previous angiography shows the M2 segment of the MCA was trifurcated after the first spontaneous recanalization. (B) The intermediate trunk was not visualized after the third infarction (arrow). MCA: middle cerebral artery

A 73-year-old woman was admitted to our hospital with a sudden onset of left hemiparesis and right conjugate deviation. The patient had a history of three cerebral infarctions caused by right MCA occlusion in the past three years and had been managed conservatively at the previous hospital. The first and second occlusions recanalized spontaneously. Neither intravenous thrombolysis nor mechanical thrombectomy (MT) was performed. Angiography at the previous hospital showed trifurcation of the M2 segment during the first and second infarctions; however, the intermediate trunk was not visualized after the third infarction (Figure 1).

**Figure 2:**
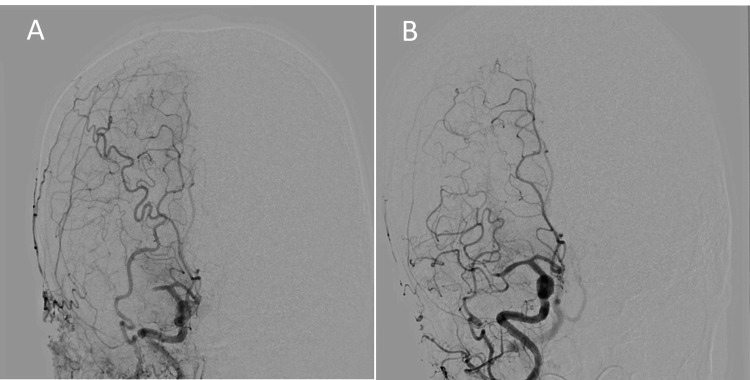
MT performed at our institution (A) Cerebral angiography performed in our hospital for the fourth ischemic stroke shows right MCA occlusion. (B) Successful recanalization could be achieved by MT using an aspiration catheter and stent retrievers. MT: mechanical thrombectomy, MCA: middle cerebral artery

Holter electrocardiography (ECG) monitoring revealed no arrhythmias, including paroxysmal atrial fibrillation, which is a well-known source of embolism. Repeated Holter ECGs were also negative for embolic arrhythmia. Transthoracic and transesophageal echocardiography revealed no intracardiac thrombi or significant aortic arch atheroma, which are potential sources of embolism. These findings supported the initial diagnosis of ESUS. The antiplatelet agents had been administered during the first and second ischemic events and were changed to rivaroxaban after the third infarction. On admission to our hospital, magnetic resonance imaging revealed an acute ischemic infarction in the right cerebral hemisphere. Cerebral angiography demonstrated right M1 segment of the MCA occlusion. The patient underwent MT with a combined technique using an aspiration catheter and stent retrievers, resulting in successful recanalization of the MCA (Figure 2).

**Figure 3:**
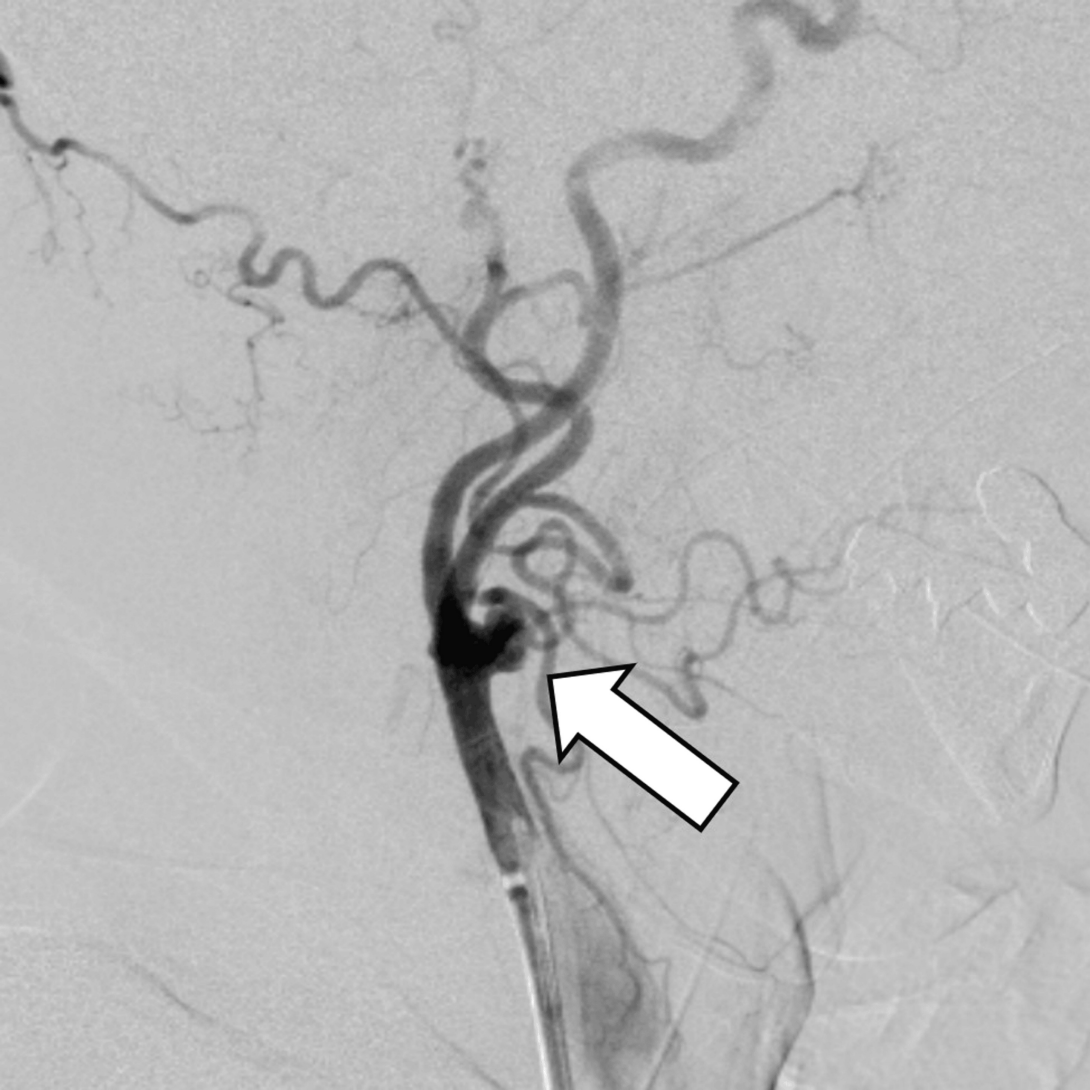
Detection of a CCA aneurysm on cerebral angiography Cerebral angiography demonstrates a right CCA aneurysm during MT (arrow). MT: mechanical thrombectomy, CCA: common carotid artery

During the MT, angiography revealed a 10 mm aneurysm at the right CCA (Figure 3).

**Figure 4:**
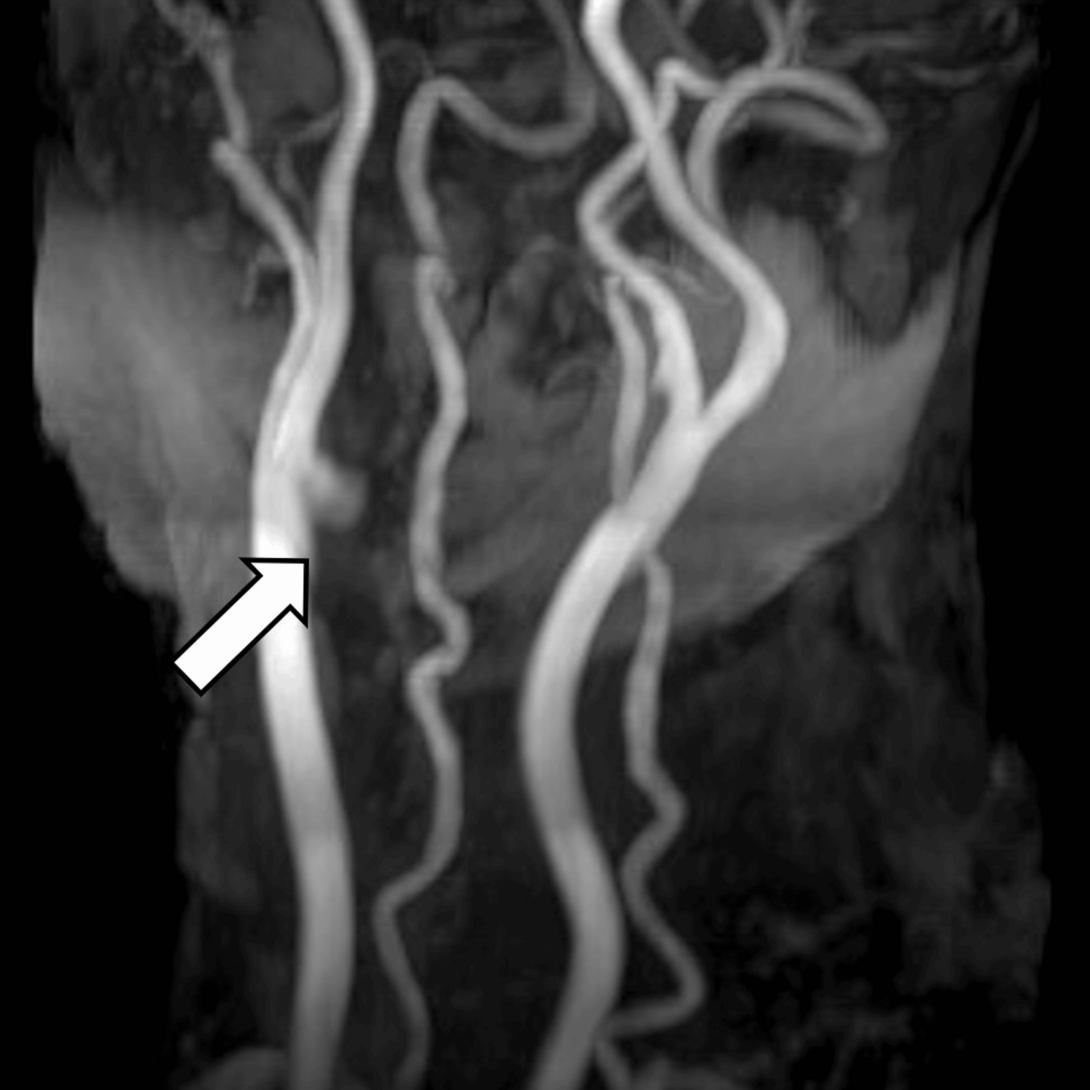
MRA at our institution MRA shows the right CCA aneurysm (arrow). MRA: magnetic resonance angiography, CCA: common carotid artery

Her left hemiparesis improved after the recanalization. Postoperative MRA also showed the right CCA aneurysm (Figure 4).

**Figure 5:**
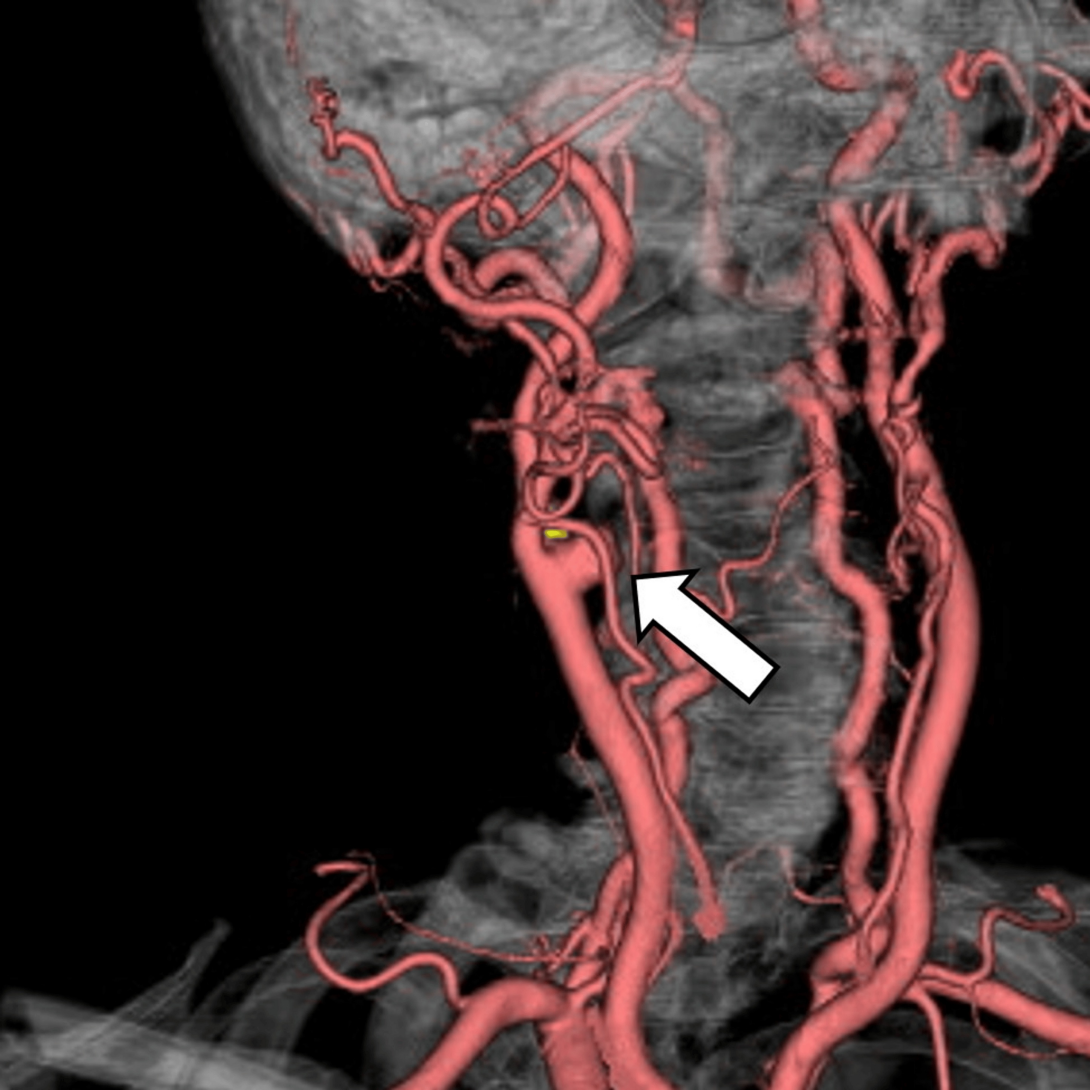
Three-dimensional CT angiography performed at the referring hospital Three-dimensional CT angiography from the referring hospital shows a previously identified aneurysm of the right CCA (arrow). CT: computed tomography, CCA: common carotid artery

In the previous hospital, the CCA aneurysm had already been detected in three-dimensional CT angiography (Figure 5).

**Figure 6:**
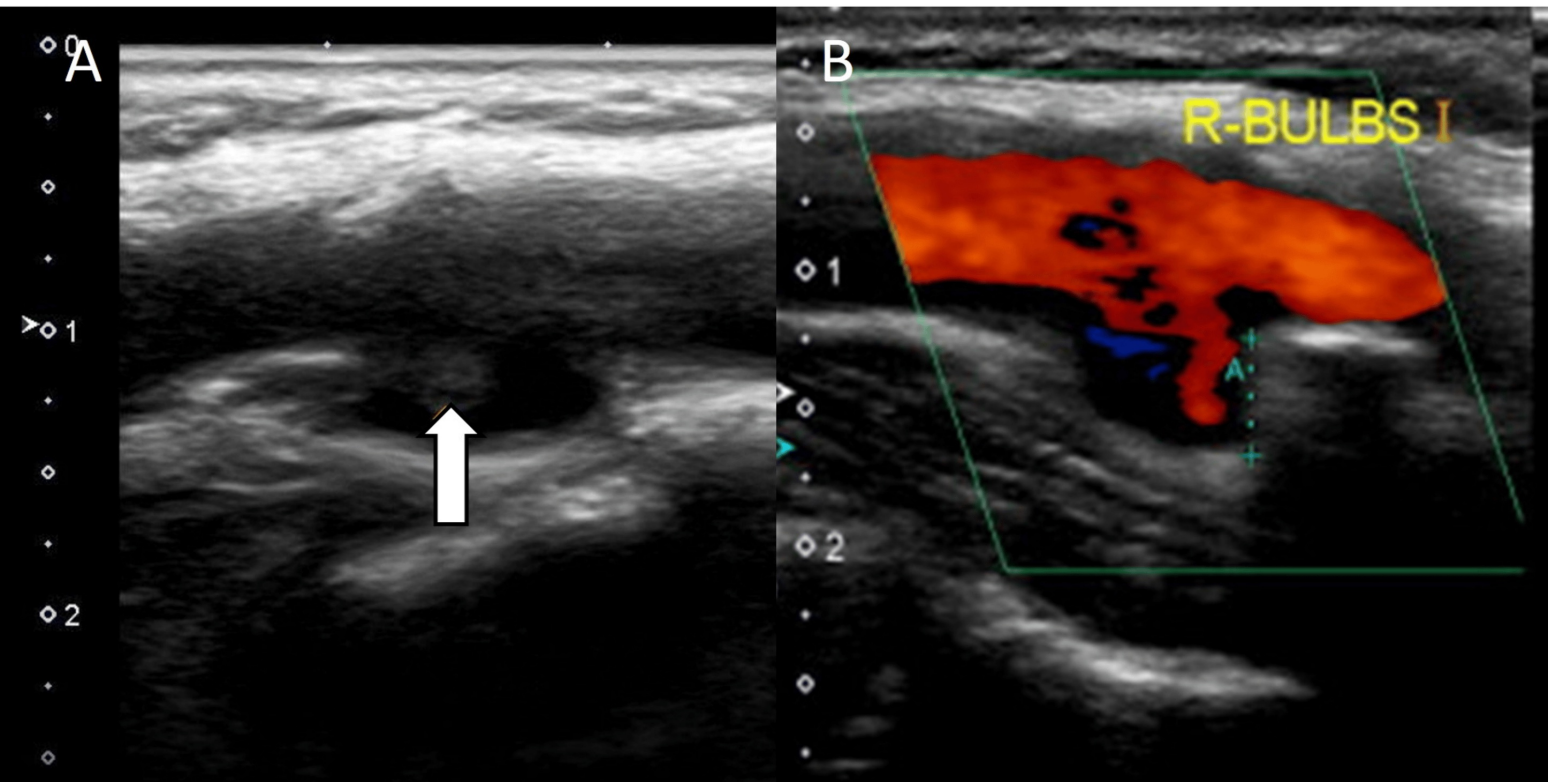
Carotid ultrasonography at our institution (A) Carotid ultrasonography reveals thrombus (arrow) in the CCA aneurysm. (B) Color Doppler imaging demonstrates turbulent flow in the aneurysm. CCA: common carotid artery

However, carotid ultrasonography showed no evidence of thrombus within the aneurysm, and it was not initially considered the embolic source. However, carotid ultrasonography performed after the MT in our hospital revealed thrombus formation within the aneurysm, and color Doppler imaging demonstrated turbulent flow (Figure 6).

**Figure 7:**
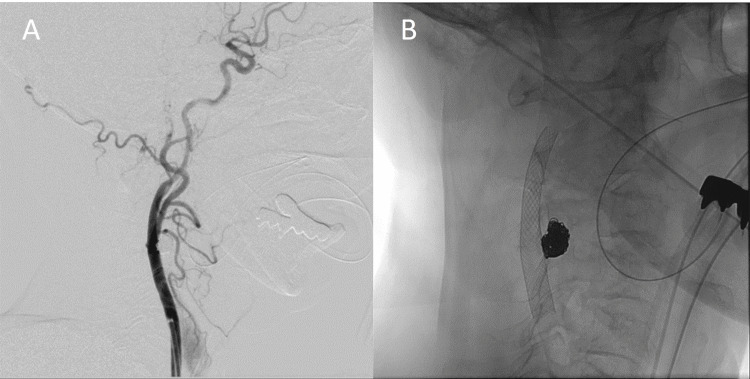
Coil embolization and carotid artery stenting for the CCA aneurysm (A, B) Closed-cell stent deployed to cover the neck of the aneurysm, and coil embolization performed using detachable coils by the jail technique. CCA: common carotid artery

Based on these findings, the thrombosed CCA aneurysm was considered the likely embolic source. After one week of dual antiplatelet therapy, intra-aneurysmal coil embolization and carotid artery stenting were performed 26 days after the MT. A microcatheter was guided into the aneurysm under proximal protection with a 9 Fr balloon guide catheter (Optimo; Tokai Medical Products, Kasugai, Aichi) and distal protection (Carotid Guardwire PS; Medtronic Vascular, Minneapolis, MN). A closed-cell stent (Carotid Wallstent, 8 × 29 mm; Boston Scientific, Marlborough, MA) was deployed to cover the aneurysm neck, and coil embolization was performed using the jail technique with detachable coils (Figure 7).

The postoperative course was uneventful, and her hemiparesis improved. Post-treatment follow-up consisted of carotid ultrasonography and MRI every six months. MRA was not suitable due to stent artifacts, but no symptomatic or asymptomatic cerebral infarction occurred. Carotid ultrasonography performed at three months postoperatively revealed no evidence of in-stent stenosis, thrombus formation, or residual aneurysm. Subsequent follow-up with MRI and carotid ultrasound showed no hemorrhagic or ischemic events. Dual antiplatelet therapy was administered for six months postoperatively, followed by single antiplatelet therapy. The patient adhered well to the medication regimen, with regular outpatient visits every two months and scheduled imaging.

Table 1 summarizes the clinical course, imaging findings, treatments, and outcomes across the four ischemic stroke events.

**Table 1:** Summary of clinical course, imaging findings, and reatments This table summarizes the patient's clinical events, neuroimaging findings, and therapeutic interventions over the course of the disease. It provides a chronological overview to help readers understand the diagnostic challenges and treatment decisions associated with recurrent embolic stroke due to a thrombosed CCA aneurysm. CCA: common carotid artery, MCA: middle cerebral artery, MT: mechanical thrombectomy

Episodes of infarction	Age (yrs)	Symptoms	Location of occlusion	Reperfusion	Medication
1 (previous hospital)	69	Left hemipalsy	Right MCA M1	Yes	Argatroban, cilostazol
2 (previous hospital)	70	Left hemipalsy	Right MCA M1	Yes	Ozagrel sodium, aspirin
3 (previous hospital)	71	Left hemipalsy	Right MCA M1	No	Argatroban, rivaroxaban
4 (present case)	73	Left hemipalsy	Right MCA M1	Yes	MT, apixaban

## Discussion

ECAAs account for only 0.4-4% of all peripheral artery aneurysms [7,8] and are located in the internal carotid artery in 35-46% of cases and the CCA in 8-42% [1,9,10]. These aneurysms are primarily caused by trauma and atherosclerosis, and other causes include pseudoaneurysm after carotid endarterectomy and vasculitis, such as Takayasu arteritis. Rare causes such as fibromuscular dysplasia have also been reported. However, in approximately 20% of cases, no specific cause is identified [1,10-12]. Approximately 87% of cases of the ECAAs are symptomatic, presenting with cerebral ischemia, cervical mass, local pain, or cranial nerve dysfunction (CND) [9,10]. Although arteriogenic embolism due to intra-aneurysmal thrombus is a known mechanism of cerebral ischemia, it might often be underrecognized in stroke etiology.

In the present case, comprehensive evaluations, including Holter ECG, carotid ultrasonography, and transthoracic and transesophageal echocardiography, were conducted at the previous hospital. However, these examinations revealed no evidence of an embolic source, including paroxysmal atrial fibrillation, atrial septal defect, or significant carotid and aortic plaque. Based on these findings, the patient was diagnosed with ESUS and was treated with antithrombotic agents. The CCA aneurysm had been identified previously, but it was not initially considered the embolic source due to the absence of visible thrombus. It is possible that turbulent flow within the aneurysm led to thrombus formation and embolic events. However, these findings were not detected on the carotid ultrasound conducted at the referring hospital. Doppler signal interpretation can be influenced by the operator’s skill and scanning conditions, and flow abnormalities may not always be consistently detected [13]. While the case was initially classified as ESUS, it ultimately revealed a structural vascular pathology that was not evident during early evaluations. This underscores the importance of repeated imaging, such as MRA and carotid ultrasonography, when assessing potential embolic sources in recurrent stroke. In patients with ESUS, it is important to maintain a high index of suspicion for ECAAs as potential embolic sources, even when thrombus is not initially evident on imaging [1,5]. Repeat vascular imaging may be necessary to detect interval changes such as thrombus formation [13]. Clinicians should consider incorporating scheduled follow-up imaging into the diagnostic strategy for patients with unexplained embolic strokes and known vascular anomalies, especially when no alternative embolic source is identified.

In this case, the aneurysm was ultimately identified as the embolic source based on the presence of thrombus and turbulent flow observed on the ultrasonography after the MT. Recurrent infarction occurred despite anticoagulation therapy, prompting the decision for endovascular intervention for the thrombosed aneurysm.

Treatment options for ECAAs include conservative management, surgical repair, and endovascular treatment [1,4,14]. Patients with symptoms of cervical mass, local pain, and CND are frequently surgically treated. Traditional surgery involves aneurysm resection with or without interposition grafting, but it carries a risk of cerebral ischemia and CND [15]. In the previous studies, surgical and endovascular treatments have both been reported to result in lower stroke recurrence in long-term follow-up [15-18]. Endovascular approaches, including stenting and coil embolization, are associated with lower morbidity and shorter hospital stays [19,20]. However, specialized departments and devices for treatment vary from country to country, so standard treatment methods cannot be established. In the present case, given the presence of recurrent ischemic events and the absence of CND, an endovascular approach was chosen. A closed-cell stent was used to prevent thrombus migration, and coil embolization was added to eliminate blood flow within the aneurysm. We considered deploying two overlapping Wallstents, but we were concerned about whether such an approach would provide sufficient flow diversion effect and whether it might increase the risk of in-stent thrombosis.

Regarding coil embolization, we were initially concerned about potential symptoms from mass effects. However, since the patient did not present with any neurological symptoms suggestive of nerve compression at the time of treatment, we prioritized the strategy of eliminating blood flow within the aneurysm to promote rapid thrombosis and prevent early stroke recurrence. Therefore, we decided to add coil embolization. Cerebral angiography after the treatment demonstrated no blood flow in the aneurysm, and no recurrence of the cerebral infarction was observed for three years. The present case suggests that endovascular treatment, including CAS and coil embolization, could be effective and safe for a thrombosed CCA aneurysm.

Several case reports have described embolic stroke caused by CCA aneurysms, but such reports remain limited in number. For example, Barlinn et al. reported a case in which an extracranial internal carotid artery aneurysm led to cerebral embolism, identified only after recurrence and repeat imaging [1,5,6]. Compared with these reports, our case is unique in demonstrating four recurrent ischemic strokes over several years, including multiple episodes of LVO, with no other embolic source identified. This highlights the diagnostic challenges in recognizing extracranial aneurysms as potential embolic sources in ESUS.

## Conclusions

This case highlights that a thrombosed ECAA can be a potential but often underrecognized source of recurrent embolic stroke. In patients with recurrent cerebral infarction and no clear embolic source, careful evaluation of the cervical arteries, including carotid ultrasonography and MRA, should be considered. Endovascular treatment with carotid artery stenting and coil embolization may be a safe and effective option for preventing further embolic events in such cases.
